# Near-normal values of extravascular lung water in children

**DOI:** 10.1186/cc10854

**Published:** 2012-03-20

**Authors:** J Lemson, C Cecchetti, A Nusmeier

**Affiliations:** 1 Radboud University Nijmegen Medical Centre, Nijmegen, the Netherlands; 2 IRCS Bambino Gesù Roma, Rome, Italy

## Introduction

Extravascular lung water (EVLW) reflects the amount of pulmonary edema and can be measured at the bedside using the transpulmonary thermodilution method (TPTD) incorporated in the PiCCO device (Pulsion, Germany). Currently, normal values of EVLW for the use in children are unavailable. This study was designed to collect near-normal values of EVLW in children after recovery from critical illness.

## Methods

In this prospective observational multicenter study (five sites), pediatric TPTD measurements were collected from children admitted to a pediatric ICU without or after resolution of pulmonary abnormalities. Inclusion criteria were minimal or no respiratory support and stable hemodynamics. We searched typically for the last lung water measurement prior to removal of the PiCCO system. EVLW was indexed using predicted body weight (EVLWI) calculated using height, based upon WHO data.

## Results

**Figure 1:**
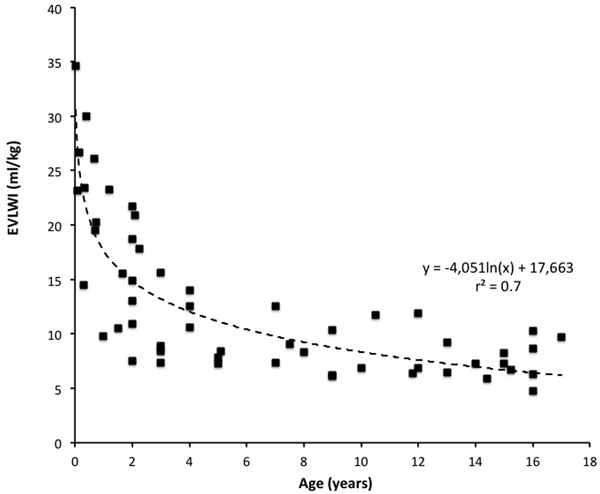


Fifty-five children aged from 0 to 16 years were included. Mean values (range) were: age 6.5 (0.04 to 16) years, weight 25.8 (3.7 to 80) kg, mean arterial blood pressure 79 (48 to 131) mmHg, PaO_2_/FiO_2 _ratio 388 (171 to 662) mmHg, cardiac index (CI) 4.5 (2.2 to 6.7) l/minute/m^2^, global end-diastolic volume (GEDVI) 490 (211 to 718) ml/m^2^, EVLWI 12.7 (4.7 to 34.6) ml/kg. Figure 1 shows the logarithmic relation between EVLWI and age with an *r*^2 ^of 0.7. There was no significant correlation between GEDVI or CI and age.

## Conclusion

Near-normal values of EVLW in children are strongly correlated with age. Based upon these data, normal values can be constructed for future clinical use.

